# HERV-H RNA is abundant in human embryonic stem cells and a precise marker for pluripotency

**DOI:** 10.1186/1742-4690-9-111

**Published:** 2012-12-20

**Authors:** Federico A Santoni, Jessica Guerra, Jeremy Luban

**Affiliations:** 1 Department of Genetic Medicine and Development, University of Geneva, 1 rue Michel-Servet, Geneva, CH-1211, Switzerland; 2 Department of Microbiology and Molecular Medicine, University of Geneva, 1211, Geneva 4, Switzerland; 3 Program in Molecular Medicine and Biochemistry & Molecular Pharmacology, University of Massachusetts Medical School, 373 Plantation Street, Biotech 2, Suite 319, Worcester, MA, 01605, USA

**Keywords:** HERV-H, Endogenous retrovirus, Pluripotency, Long non-coding RNA, Embryonic stem cell, Induced pluripotent stem cell

## Abstract

**Background:**

Certain post-translational modifications to histones, including H3K4me3, as well as binding sites for the transcription factor STAT1, predict the site of integration of exogenous gamma-retroviruses with great accuracy and cell-type specificity. Statistical methods that were used to identify chromatin features that predict exogenous gamma-retrovirus integration site selection were exploited here to determine whether cell type-specific chromatin markers are enriched in the vicinity of endogenous retroviruses (ERVs).

**Results:**

Among retro-elements in the human genome, the gamma-retrovirus HERV-H was highly associated with H3K4me3, though this association was only observed in embryonic stem (ES) cells (p < 10^-300^) and, to a lesser extent, in induced pluripotent stem (iPS) cells. No significant association was observed in nearly 40 differentiated cell types, nor was any association observed with other retro-elements. Similar strong association was observed between HERV-H and the binding sites within ES cells for the pluripotency transcription factors NANOG, OCT4, and SOX2. NANOG binding sites were located within the HERV-H 5^′^LTR itself. OCT4 and SOX2 binding sites were within 1 kB and 2 kB of the 5^′^LTR, respectively. In keeping with these observations, HERV-H RNA constituted 2% of all poly A RNA in ES cells. As ES cells progressed down a differentiation pathway, the levels of HERV-H RNA decreased progressively. RNA-Seq datasets showed HERV-H transcripts to be over 5 kB in length and to have the structure 5^′^LTR-*gag*-*pro*-3^′^LTR, with no evidence of splicing and no intact open reading frames.

**Conclusion:**

The developmental regulation of HERV-H expression, the association of HERV-H with binding sites for pluripotency transcription factors, and the extremely high levels of HERV-H RNA in human ES cells suggest that HERV-H contributes to pluripotency in human cells. Proximity of HERV-H to binding sites for pluripotency transcription factors within ES cells might be due to retention of the same chromatin features that determined the site of integration of the ancestral, exogenous, gamma-retrovirus that gave rise to HERV-H in the distant past. Retention of these markers, or, alternatively, recruitment of them to the site of the established provirus, may have acted post-integration to fix the provirus within the germ-line of the host species. Either way, HERV-H RNA provides a specific marker for pluripotency in human cells.

## Background

Vertebrate genomes contain retroviral sequences that are believed to be remnants of exogenous retroviral infection from the distant past [1]. The genesis of these endogenous retroviruses (ERVs) necessitates establishment of provirus by the ancestral, exogenous retrovirus within host germ cells, such that these elements are maintained as heritable genetic elements in the host species. Human endogenous retroviruses (HERVs) are not uncommon, and account for at least 8% of the total human DNA. By comparison with DNA sequences from exogenous retrovirus families, three main classes of HERVs, gamma, beta, and delta have been identified [2]. Phylogenetic analyses identified HERV-K, a betaretrovirus, and HERV-H, a gammaretrovirus, as the most recent entries into the genomes of primates, 10 and 25 million years ago, respectively [3,4].

Though some HERVs are transcriptionally active, most retroviral sequences in the human genome are corrupted by mutations or successive insertion of transposable elements; most HERVs lack intact open reading frames for viral protein production and no autonomously replicating HERV has been identified. As a result, HERVs are generally considered non-functional, junk DNA. In some cases, though, HERVs make significant - even essential - contributions to the normal physiology of the host species [5-8]. HERVs have also been implicated in the development of pathological conditions [9-14]. Interestingly, HERVs can be activated by exogenous retrovirus infection [15] and, in reciprocal fashion, the immune response to these elements can influence the outcome of infection by pathogenic, exogenous retroviruses such as HIV-1 [16,17].

Exogenous retroviruses integrate into locations throughout the host chromosomal DNA in a quasi-random fashion (reviewed extensively in [18]). Previously, we developed statistical tools to identify association between the sites of provirus establishment and chromosome marks – as determined by chromatin immuno-precipitation with massively parallel DNA sequencing (ChIPSeq) [18,19]. From this analysis we identified chromatin modifications (H3K4me3, H3K4me1, and H3K9ac) and binding sites for transcription factors (STAT1) that predict the site of integration for gamma-retroviruses with great accuracy in a cell-type specific manner [18,19]. Precise markers such as these were not identified for other classes of retrovirus such as the lentivirus HIV-1 [18]. Here, the same statistical methods were exploited to determine whether any ERVs retain association with cell type-specific chromatin features that might have determined the site of integration in the distant past, or that served to fix the provirus in the genetic patrimony of the host species.

## Results

### Search for chromatin features near the site of ERVs

Previously observed, high-level association between gamma-retrovirus integration sites and particular epigenetic markers [18] prompted a quest to find association between any known endogenous retroviral element in the human and mouse genomes, and the cell-type specific localization of particular chromosomal features. For this purpose, ChIPSeq profiles were evaluated from more than 40 different human and mouse cell types, including ES cells, iPS cells, monocytes, HeLa cells, CD4+ T cells, and CD34^+^ hematopoietic cells (Table 1). H3K4me3 was used in the initial analysis because of the availability of ChIPSeq datasets from a large number of cell types for this marker. The ERV dataset was compiled using all endogenous, LTR-containing elements annotated via RepeatMasker on the human reference genome hg18 (UCSC) or the mouse reference genome mm9 (UCSC).

**Table 1:** Data used for analysis of endogenous retroviruses

**Data type GEO accession number**	**Factor**	**Cell type or tissue**	**Reference**
**Human**			
ChIPSeq, GSE16256	H3K4me3, H3K27me3	H1, H9, I3, iPS, IMR90	[20]
ChIPSeq, GSE22499	H3K4me3	BG01/03, WIBR1/2/7, hiPSA6/C1, Fibroblast	[21]
ChIPSeq, GSE15353	H3K4me3	HeLa w/o IFN	[22]
ChIPSeq, GSE19465	H3K4me3	hiPS-11a/18c/15b/20b, duodenum mucosa, BM-MSC,smooth muscle, adult liver, fetal lung, fetal brain, CD34, CD3, CD15, CD19, Pancreatic Islets	[23]
ChIPSeq, GSE20650	NANOG,OCT4, KLF4	H1	[24]
ChIPSeq, GSE18292	SOX2	H1	[25]
RNASeq, GSE23316		H1, HeLa, K562	UCSC ENCODE Project
RNASeq, GSE20301		H1, differentiated H1	[26]
**Mouse**			
ChIPSeq, GSE22075	Mouse H3K4me3	ES, LSK cells	[27]
ChIPSeq, GSE12241	Mouse H3K4me3	ES, MEF	[28]

**Figure 1:**
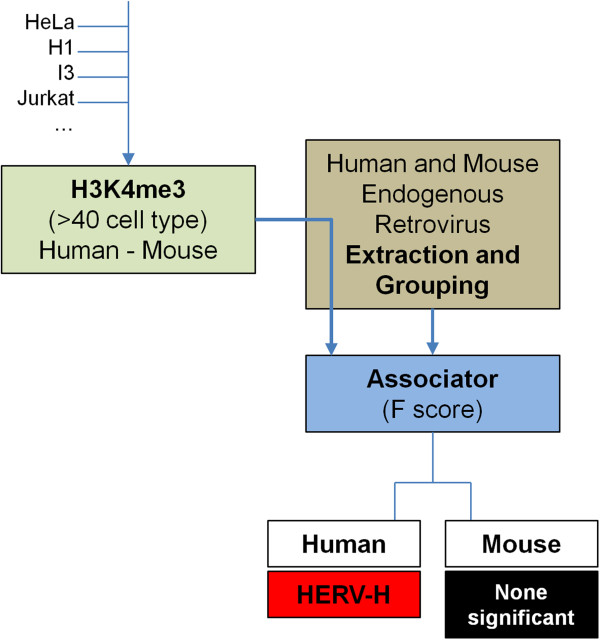
**Schematic showing the strategy for detecting association between endogenous retro elements and the H3K4me3 marker.** H3K4me3 CHiPSeq data for more than 40 different cell types were checked for association within 2 kB of all annotated endogenous retroviral elements (hg18) using methods described in reference [18]. Only HERV-H proviruses showed association with H3K4me3, and this was only significant in human ES cell lines. No association between H3K4me3 and endogenous retro-elements was detected in the mouse.

Figure 1 shows this analysis schematically. The block *Associator* is a computational module fed by ChIPSeq profiles and LTR loci. Among all LTRs, only HERV-H showed a significant association with H3K4me3, though this association was only with some human cell types; the location of the endogenous gamma-retrovirus was associated with H3K4me3 profiles in ES cells (F-score >0.8; p < 10^-300^, by Fisher exact test) and, to a lesser extent, in iPS cells (F-score >0.7; p < 10^-100^). F-scores > 0.5 are considered significant with 1.0 maximal [18], so these values are highly significant. In contrast, no retroviral element in the mouse was found to be associated with H3K4me3.

**Figure 2:**
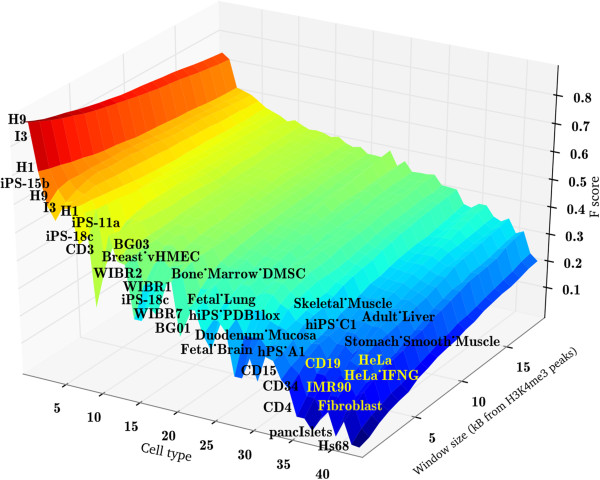
**H3K4me3 association with HERV**-**H proviruses, as measured with the F score, in 40 different cell types, as a function of the distance from the marker.** The F score is reported on the z-axis. Cell types are listed on the x-axis, while the window size is reported on the y-axis. Yellow to red bands indicate a significant association (F score >0.5). Significance decreases as the color shifts from red to blue. 12 out of 15 of the cell types with significant F scores are either human ES cells or iPS cells.

**Figure 3:**
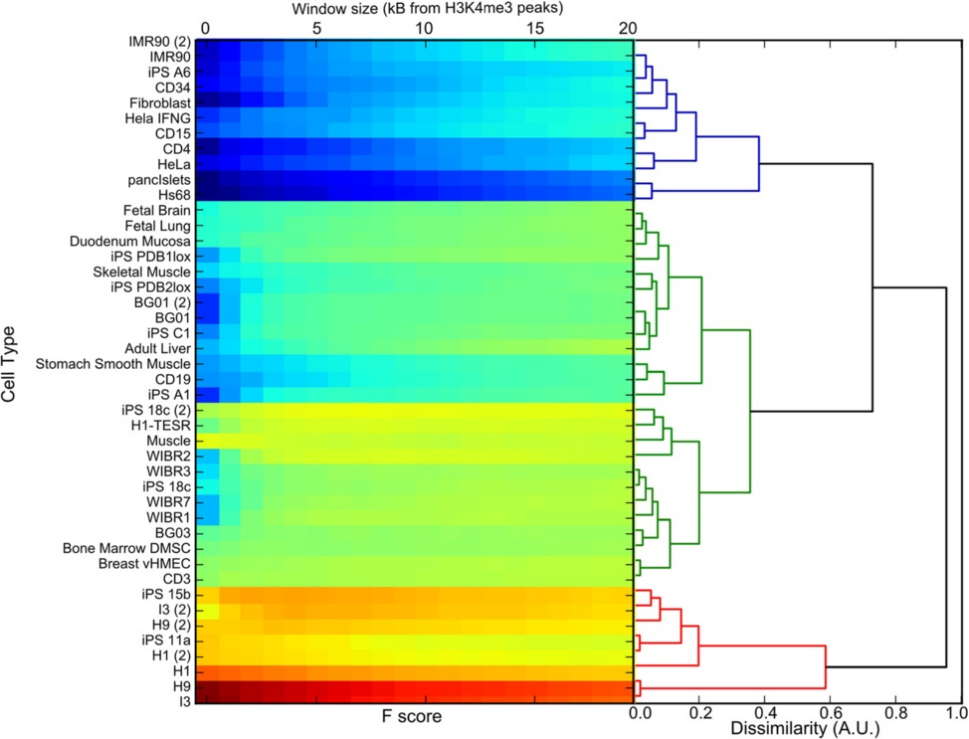
**Hierarchical cluster analysis of the HERV-H/H3K4me3 F score, as a function of the window size (horizontal axis).** F score is represented using the color scale as in Figure 2. The highly associated cluster (red tree) was made of human ES and iPS cells. The cluster with medium association (green) consisted of mesenchymal stem cells (DMSC), fetal lung and brain cells, and some iPS cells, with F scores that were barely significant. The blue cluster consisted of differentiated cells and the mean F score of 0.36 was insignificant.

**Figure 4:**
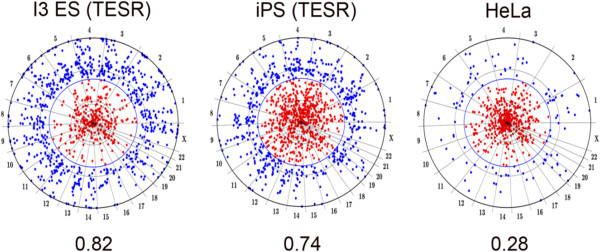
**Chromosome projection mandalas showing the proximity of each HERV-H provirus to the nearest site of H3K4Me3 on the chromosome, in human I3 ES cells, iPS-15b cells, and HeLa cells.** Each dot on the mandala indicates an HERV-H provirus, as described in reference [18]. The angular distance around the mandala indicates the linear position of each provirus on the indicated chromosome. The radial distance from the perimeter indicates the distance of the provirus from the nearest H3K4Me3 site, in log scale from 0 to 1 megabase. Blue dots are HERV-H proviruses within 2 kB from the nearest marker. Red dots are proviruses >2 kB away from the nearest H3K4Me3 site. The association strength (F score) is written under each Mandala. F score > 0.5 constitutes a significant association.

The data were assessed by means of Hierarchical Clustering. Each association profile was calculated as a function of the distance between HERV-H and the nearest H3K4me3 marker, discretized with steps of 0.5 kB. The specific cells are listed on the x axis of Figure 2, and the window size in kilobases is shown on the y-axis. The Euclidean distance between these profiles was used to discriminate among clusters. The algorithm identified three main clusters of high, medium and low association (represented in red, green and blue in Figure 3). The cluster with the highest association (red) was populated by ES cells (H1, H9 and I3) and iPS cells (iPS-15b and iPS-11a), with a mean F score of 0.74. The cluster with medium association (green) was populated by bone marrow mesenchymal stem cells (DMSC), breast cancer cells (vHMEC), fetal lung cells and fetal brain cells; it had F scores that were just barely significant. Some iPS cells (A6, PDB1lox, 18c, C1) fall within this cluster, thus confirming that many iPS cells have epigenetic profiles distinct from those of ES cells [29]. The blue cluster consisted of differentiated cells, including CD4^+^ T cells, fibroblasts, pancreatic islet cells, and HeLa cells; the mean F score of 0.36 was insignificant. The association of HERV-H with the post-translational histone modification H3K4me3 correlated strongly with the degree of cell differentiation, a relationship that was clearly visible using chromosome projection mandalas (Figure 4).

### HERV-H expression in ES cells

**Figure 5:**
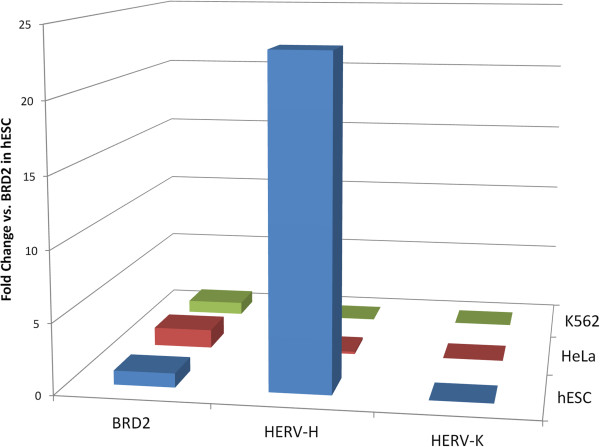
**Cumulative expression of all HERV-H proviruses in human H1 ES cells (hESC), HeLa cells, or K562 cells, compared to expression of HERV-K and BRD2, a constitutive gene with the same expression level in all three cell types.** In human ES cells, HERV-H is expressed 1000-fold higher than HERV-K and 25-fold higher than BRD2. HERV-H expression is barely detectable in HeLa, and no significant HERV-H expression was detected in K562 cells. RNASeq data for this analysis were from reference [33].

Given the remarkable, pluripotent stem cell-specific association of HERV-H with H3K4me3, a marker for transcriptionally active promoters [30,31], HERV-H expression would be expected to be higher in ES and iPS cells than in differentiated cell types. Consistent with this possibility, HERV-H was not associated with H3K27me3 (F score 0.2), a marker for transcriptional repression in ES cells [32]. ENCODE Project RNA-seq data sets [33] of paired 75 nucleotide reads from human ES cells and 6 differentiated cell types were assessed for HERV-H RNA peaks. In H1 human ES cells, HERV-H RNA accounted for 2% of the total RNA. This extraordinary level of HERV-H expression was confirmed with RNA-seq data [34] from H9, another ES cell line. For comparison, expression of the younger and better-conserved beta-retrovirus HERV-K was 1000-fold lower than HERV-H in H1 ES cells. HERV-H RNA was 100-lower in HeLa cells and more than 100-fold lower in K562 myelogenous leukemia cells, GSM12878 lymphoblastoid cells, HepG2 hepatocellular liver carcinoma cells, human umbilical vein endothelial cells (HUVEC), and NHEK epidermal keratinocytes. BRD2, a gene that is expressed at nearly the same level in all these cell types was 25-fold lower in expression than HERV-H in ES cells (Figure 5).

**Figure 6:**
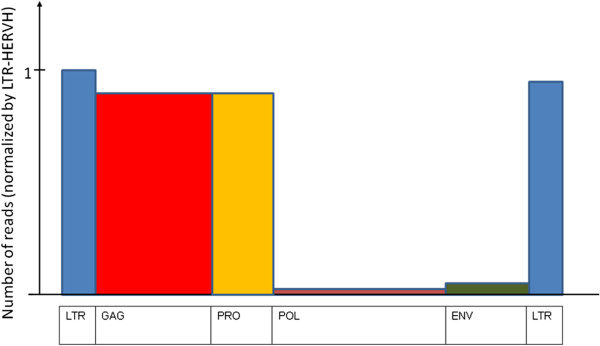
**Mapping of RNA-seq reads from H1 human ES cells on a schematic of the HERV-H provirus.** The quantity of each RNA read was normalized to the reads corresponding to the 5^′^ LTR. Only RNA fragments corresponding to 5^′^LTR-*gag*-*pro*-3^′^LTR were expressed to a significant level in human ES cells.

Almost all the HERV-H RNA expressed in ES cells had the structure 5^′^LTR-*gag*-*pro*-3^′^LTR with deletion of the *pol* and *env* regions, and no intact open reading frames. HERV-H fragments containing *pol* and *env* sequences were barely detected (Figure 6).

**Figure 7:**
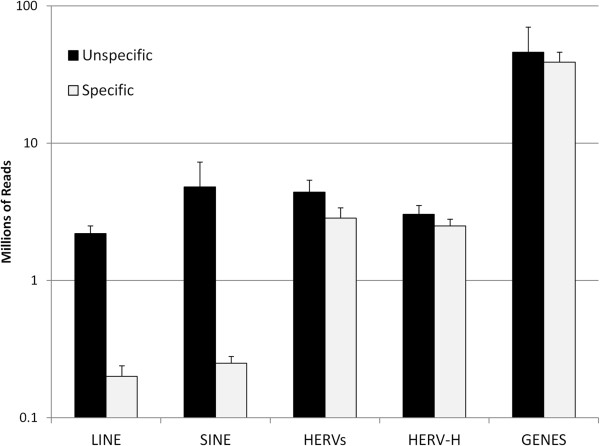
**HERV-H expression accounts for nearly all HERV expression in human ES cells and is a not a non-specific consequence of wide-spread hypo-methylation in these cells.** Quantitation of the RNA-seq reads from H1 ES cells, broken down according to the LINES, SINES, all HERVs, the nearly 1,000 HERV-H proviruses, and conventional genes. HERV-H RNA accounted for nearly all the HERV RNA in human ES cells, and 2% of total RNA. Specific vs. non-specific expression was determined by comparing the expression level of each element to the surrounding sequences.

It has been reported that human ES cell DNA is hypo-methylated with respect to differentiated cell lines, and that this global effect releases all endogenous elements, such as SINEs, LINEs, and HERVs, from transcriptional silencing [35]. To determine if HERV-H expression in ES cells is simply a result of this global trend, the RNA-seq data was used to compare the expression level of all repetitive elements in ES cells. Compared to LINEs and SINEs, HERV-H was by far the most expressed repetitive element in human ES cells, accounting for nearly all transcription of the HERV family (Figure 7).

It is also important to consider that expression of endogenous retro-elements might be influenced by local effects from adjacent transcriptional units. The difference between the expression level of the repetitive elements and the expression level of the surrounding sequences was assessed (see Methods for details). By this measure, HERV-H exhibited transcriptional specificity comparable to that of conventional genes, in terms of specific/unspecific transcription ratio (0.85 vs. 0.86), while LINEs and SINEs had a transcription ratio of 0.06 and 0.03, respectively (Figure 7).

### HERV-H expression over the course of human ES cell differentiation

**Figure 8:**
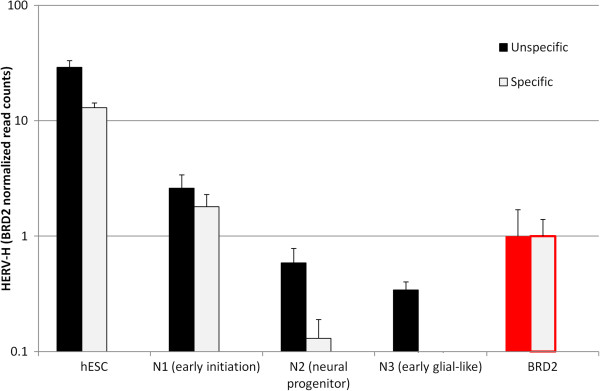
**HERV-H expression correlates with differentiation status.** HERV-H expression as H1 human ES cells differentiate down a pathway towards neural progenitors and early glial cells. Black bars indicate unspecific expression; white bars represent specific expression, adjusted for expression as described in Figure 7. BRD2 has the same expression level at each stage of differentiation and was used to normalize HERV-H RNA levels.

The disparity between the high transcriptional level of HERV-H in human ES cells, and the near absence of HERV-H transcription in differentiated cells prompted an assessment of HERV-H expression as ES cells differentiate. To accomplish this, raw data was analyzed from a 4-point time course experiment (http://www.ncbi.nlm.nih.gov/geo/query/acc.cgi?acc=GSE20301) in which RNA was collected from human ES cells in the undifferentiated state (N0), early initiation (N1), neural progenitor (N2), and pre-glial cell (N3) stages [26]. High HERV-H transcriptional levels were confirmed in the undifferentiated N0 stage, and HERV-H levels decreased progressively with a magnitude that correlated with the differentiation time-point (Figure 8).

### HERV-H and pluripotency transcription factors

**Figure 9:**
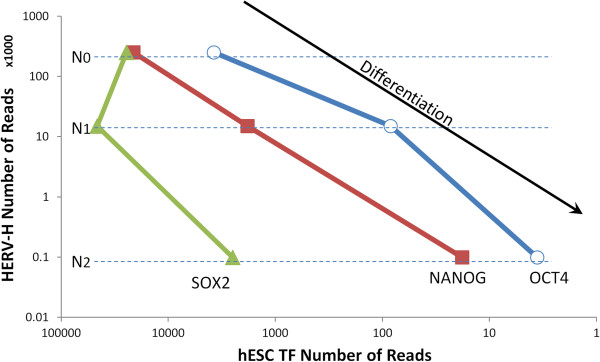
**HERV-H expression levels correlate with those of pluripotency transcription factors NANOG and OCT4 as human ES cells move down a differentiation pathway.** N0, undifferentiated ES cells. N1, early initiation stage of differentiation. N2, neural progenitor stage. OCT4 and NANOG are positively correlated with HERV-H (ρ = 0.95, ρ = 0.84, respectively). SOX2 shows no correlation.

When ectopically expressed in particular combinations, the transcription factors NANOG, OCT4, and SOX2 are capable of reprogramming mature somatic cells into pluripotent stem cells [36-39]. Conversely, the expression of these factors decreases as cells differentiate [40]. The RNA-seq data sets utilized above that measure the dynamics of RNA in ES cells as they differentiate into neural progenitors was analyzed for expression of HERV-H and for these transcription factors. HERV-H RNA levels correlated well with those of NANOG and OCT4 (Figure 9). SOX2 was more stably expressed during differentiation than was NANOG or OCT4 and its expression did not correlate with that of HERV-H.

**Figure 10:**
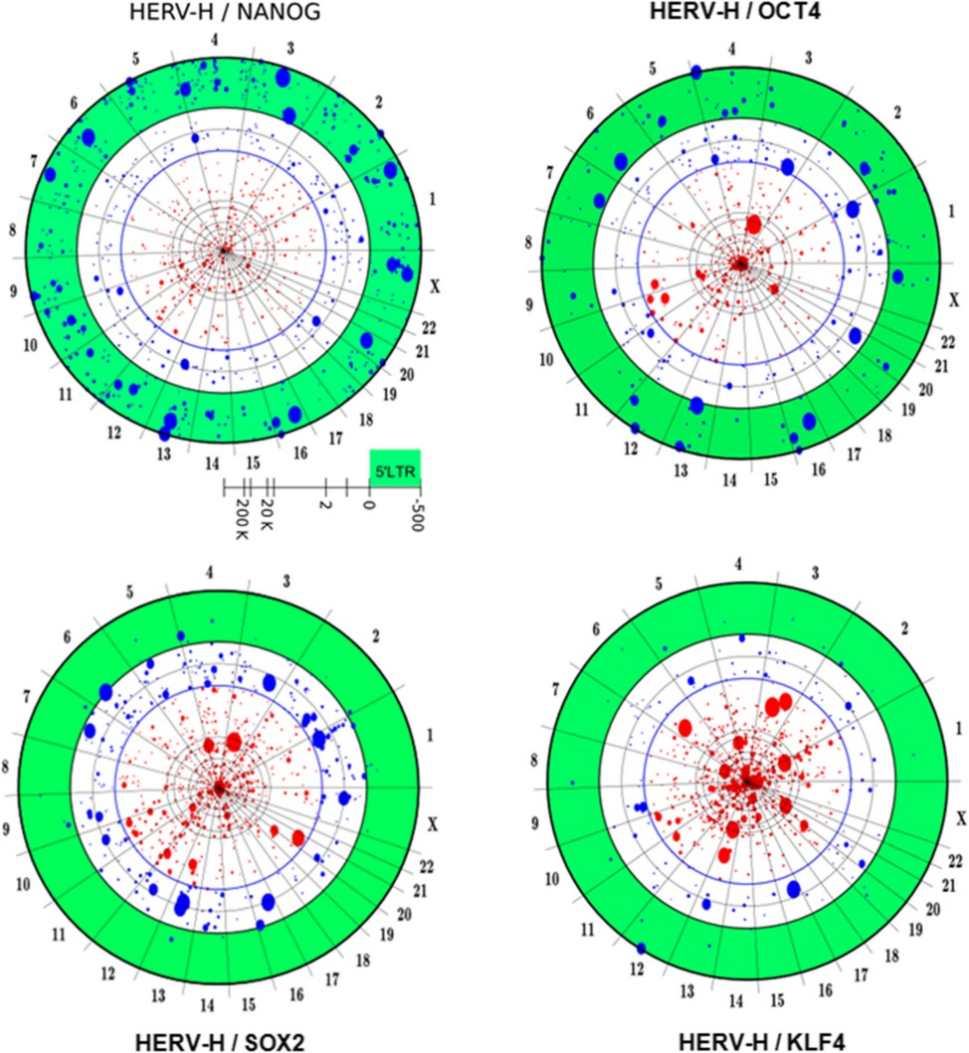
**Modified chromosome projection mandalas depicting NANOG, OCT4, SOX2 and KLF4 associations with HERV-H proviruses.** Mandalas were generated as described in reference [18] and Figure 4 above. The green ring indicates the HERV-H 5^′^ LTR region (500 nucleotides from the transcriptional start site). Dot size is proportional to the expression level of each single provirus. NANOG is bound to the 5^′^LTR of almost all highly expressed retroviruses. OCT4 shows a significant association. SOX2 binds all expressed HERV-H in a region between 1 KB and 2 KB while KLF4 does not show any significant association pattern.

To explore the possibility that these pluripotency transcription factors are somehow involved in HERV-H transcription, an association analysis was conducted between HERV-H locations and the ChIPSeq binding profiles of NANOG, OCT4, SOX2, and KLF4 extracted from ES cells [24,25] (Figure 10). Over a 2 kB window, NANOG showed the most significant association (F score 0.75, p < 10^-180^). OCT4 weakly associated with HERV-H (F score 0.55, p < 0.0001), while SOX2 and KLF4 were not associated (F score 0.46 and 0.24, respectively).

**Figure 11:**
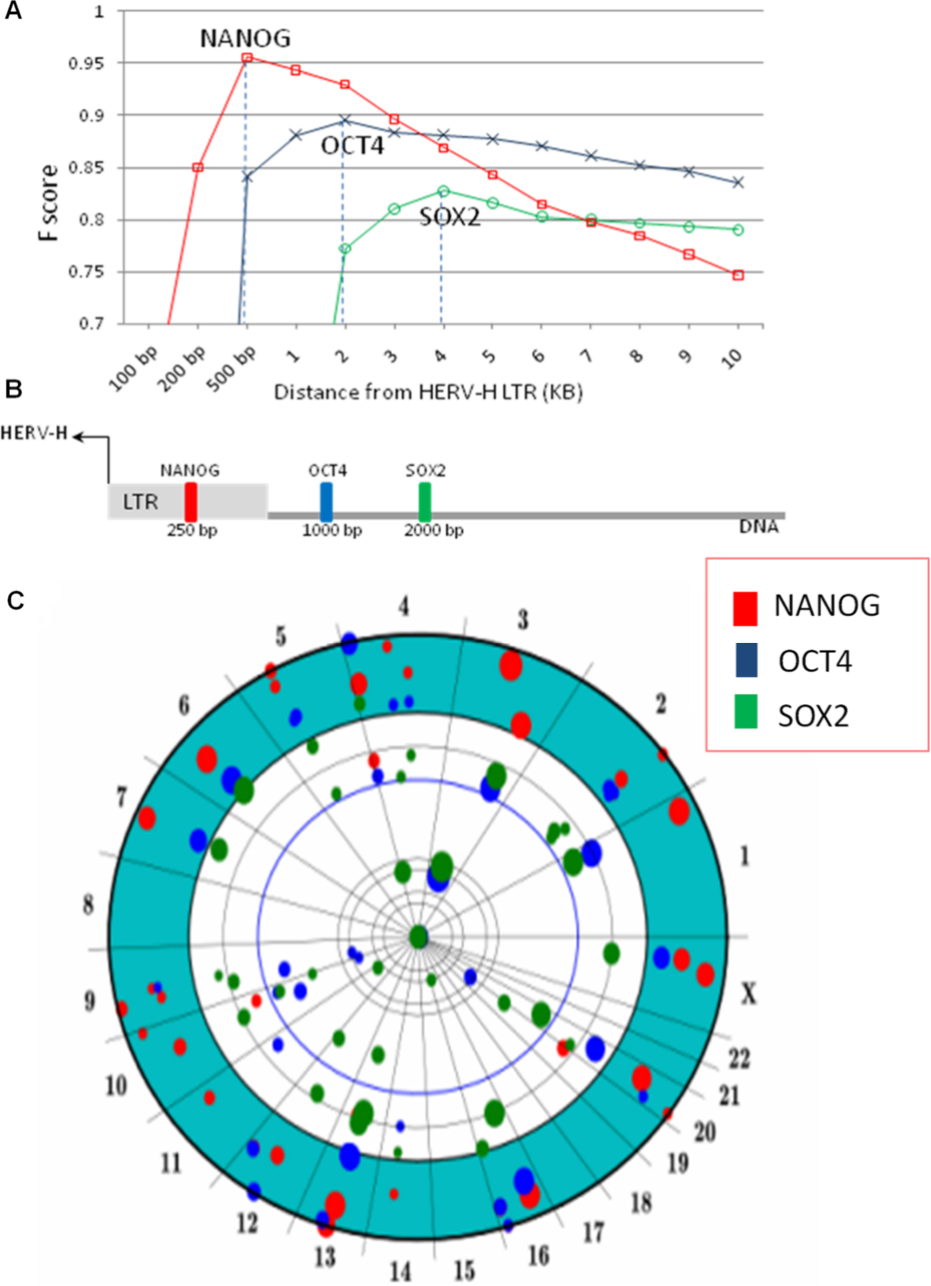
**Ordered spacing of pluripotency transcription factors, binding to the HERV-H 5^′^ LTR in human ES cells.** (**A**) Association strength (F score) of NANOG, OCT4 and SOX2 binding sites with the 50 most highly expressed HERV-H proviruses (accounting for 80% of total HERV-H expression), as a function of distance from the HERV-H transcription start site (TSS). Maxima in F score indicate the distance of greatest association. (**B**) Average distance of NANOG (red), OCT4 (blue) and SOX2 (green) to HERV-H TSS is shown schematically. As expected from a uniform distribution model, the average distance is half of the distance between maximal association and TSS. (**C**) Chromosome Projection Mandala combining NANOG, OCT4 and SOX2 with respect to 50 HERV-H proviruses. The three embryonic transcription factors bind with the same order (NANOG-OCT4-SOX2) to the promoter region of the most expressed HERV-Hs.

Statistical significance increased considerably when the expression level of individual HERV-H proviruses was monitored (Figure 11A and B). As visualized on the chromosome projection mandala in Figure 11C, NANOG binds to the LTRs of 96% of the 50 most highly expressed HERV-H proviruses. These 50 proviruses account for 80% of all HERV-H RNA. The LTR region corresponding to the first 0.5 kB of HERV-H proviral sequence is highlighted in green, with dot sizes proportional to expression levels. With a window of 0.5 kB, the F score for NANOG was nearly perfect (0.97, p < 10^-300^). Limited to this subset of HERV-H proviruses the F scores for OCT4 (0.90 wi2kB, p < 10^-300^) and SOX2 (0.85 wi4kB; p < 10^-300^) were also tightly linked to HERV-H. KLF4, in contrast, was not associated with HERV-H (F score 0.34). The average distance of the NANOG, OCT4 and SOX2 ChIPSeq peaks from the HERV-H transcription start sites was 250 basepairs, 1 kB and 2 kB, respectively, as if these factors were uniformly distributed along the HERV-H promoter in human ES cells (Figure 11B). Indeed, for 80% of the highly expressed HERV-H proviruses NANOG, OCT4 and SOX2 map in this order.

### Association of LINE elements with NANOG in human ES cells

**Figure 12:**
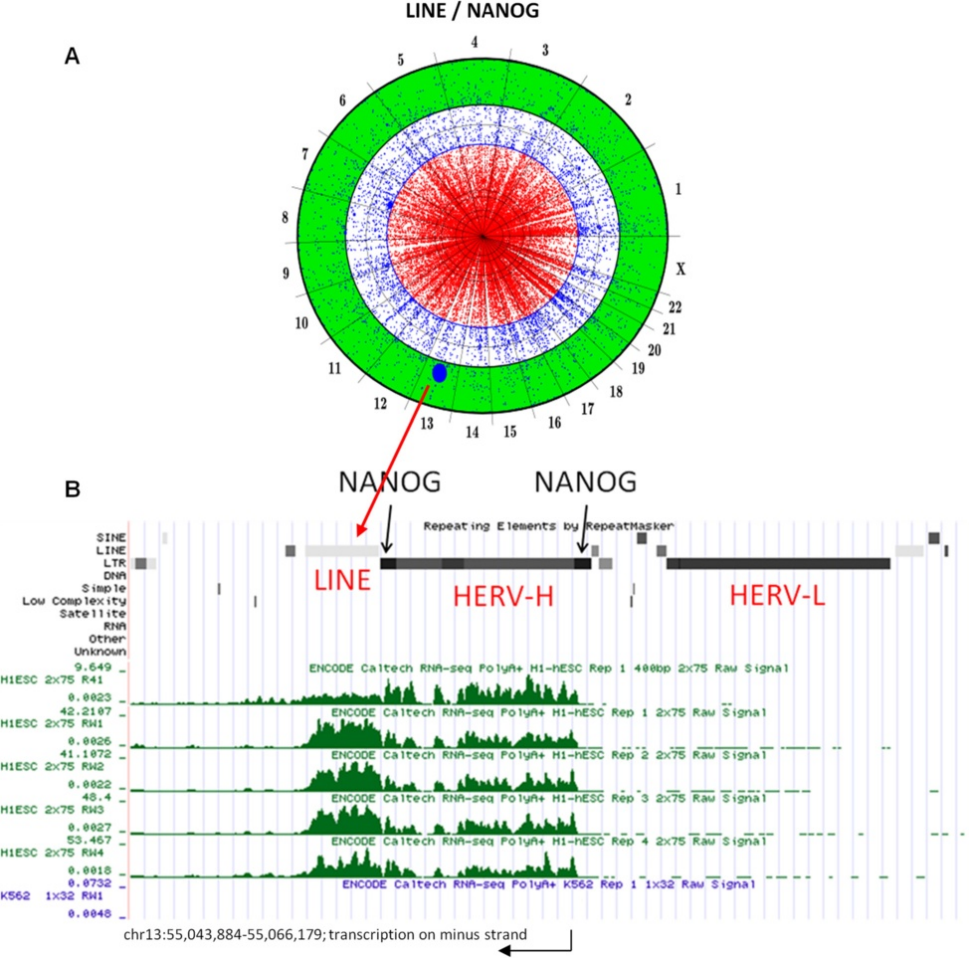
**(A) Chromosome projection mandala depicting the association between NANOG and expressed LINEs in human ES cells.** Only one LINE (large blue dot on chromosome 13) was expressed to high level in these cells. (**B**) The genomic region around the LINE on chromosome 13 is shown with the UCSC Genome Browser, where the linear chromosome is mapped on the horizontal axis. The position of the adjacent HERV-H and HERV-L proviruses is shown. Four biological replicates confirm HERV-H and LINE expression in human ES cells while no expression was detected in K562 cells (only one of the 4 replicates is shown). The direction of transcription was determined by strand-specific sequencing [33]. NANOG bound to both LTRs (represented with black squares along the LTR row) in the adjacent HERV-H. The adjacent HERV-L was not transcribed.

As a matter of comparison, a chromosome projection mandala was created in which NANOG binding sites in ES cells were compared with the location of the LINEs (Figure 12). Only one LINE, located on chromosome 13 between nucleotides 55,048,158 and 55,050,476, was expressed to an appreciable level. Interestingly, a deeper inspection of this region revealed a particular genomic structure where LINE transcription was driven by an adjacent HERV-H, bounded by NANOG binding sites at both LTRs. This element does not have an intact ORF, and its functional role is unclear. It is also noteworthy that the adjacent HERV-L is not expressed, even if it is immediately adjacent to the transcribed region, confirming that HERV-H expression is not a non-specific consequence of hypomethylation in ES cells.

## Discussion

This study discovered a strong association between the genomic location of HERV-H proviruses and H3K4me3-modified histones, that is exclusive to human ES and some iPS cells. Consistent with H3K4me3 serving as a marker for active transcription, HERV-H expression was high in these pluripotent stem cells. Moreover, the pluripotency transcription factors NANOG, OCT4 and SOX2 bound to the LTRs of transcriptionally active HERV-H proviruses, or within 2 kB of them. NANOG, OCT4 and SOX2 frequently co-occupy the promoters of target genes, many of which are transcription factors that regulate development such as homeodomain proteins [41].

These observations strongly support the hypothesis that HERV-H transcripts play a role in human pluripotency and that this role is finely regulated by three of the most important transcription factors in ES cells. In addition to the binding of NANOG, OCT4, and SOX2 to the HERV-H promoter, HERV-H RNA decreased as ES cells differentiated, in a manner that was proportional to the expression of NANOG and OCT4. Conversely, HERV-H RNA was undetectable in primary fibroblasts but increased enormously after forced re-programming to generate pluripotent stem cells (unpublished data provided by Audrey Letourneau and Stylianos Antonarakis). HERV-H, then, can be exploited as a reliable marker of ES cell pluripotency, as well as an indicator of the degree of “stemness” of iPS cells as they are generated from fibroblasts.

HERV-H transcripts are 5 to 6 kB in length and lack open reading frames. We can only speculate about the function of these lncRNAs. They might, for example, serve to soak-up miRNAs that promote differentiation, as has been shown with linc-MD1 in muscle differentiation [42] or the *PTENP1* pseudogene in the regulation of PTEN and growth suppression [43]. They might bind to chromatin and act as a scaffold for the local recruitment of pluripotency transcription factors, similar to other lncRNAs like HOTAIR for histone modification complexes [44] and Xist in the context of X-chromosome inactivation [44-46]. Alternatively, HERV-H might counteract retrovirus spread by interfering with packaging of retroviral genomic RNA [47,48] or by soaking up miRNAs that are required for retrovirus transduction.

The study here failed to identify chromatin markers that associate with endogenous retro-elements in mice. This was somewhat surprising given the many endogenous retro-elements in this species, including endogenous gamma-retroviruses, some of which are intact and functional [8]. It was also surprising because exogenous gamma-retroviruses have the same integration site preferences in mouse cells as they have in human cells [18]. MLV integration sites are associated with the H3K4me3 profile in mouse embryonic fibroblasts (F score = 0.83; p < 10^-100^). Similar results with murine hematopoietic stem cells (F score of 0.81; p < 10^-100^) indicate that, as in human cells, the association strength is cell-type dependent.

One possible explanation for the failure to identify chromatin markers associated with endogenous murine retroviruses is species-specific differences in the recruitment of the transcriptional silencing machinery. In murine ES cells, for example, a sequence-specific DNA-binding protein, ZNF809 [49], recruits TRIM28 and other components of the cellular machinery that silence MLV [50]. ZNF809 has no orthologue in humans; perhaps ZNF809 arose as a result of selective pressure exerted by murine specific gamma-retroviruses during evolution.

Previous work demonstrated that when exogenous retroviruses integrate they home to sites of H3K4me3 [18]. Similarly, the association of endogenous gamma-retrovirus HERV-H with H3K4me3 suggests that when human and simian germ cells were bombarded with the HERV-H ancestor 15 to 30 millions years ago, these ancient retroviruses integrated in proximity to H3K4me3-marked chromatin. These proviruses might then have retained these cell-type specific marks as they became fixed in the primate genome. Alternatively, unmethylated HERV-H LTRs might have recruited chromatin remodeling factors and induced H3K4me3 modification of the viral promoter after integration had occurred.

Analysis of the DNA surrounding HERV-H proviruses failed to clarify which of these two scenarios is more likely. Additionally, search for epigenetic markers like H3K4me3 in syntenic regions in the mouse genome was attempted to determine if these chromatin marks are conserved across the species and predate the entry of HERV-H into the primate genome. DNA surrounding HERV-H proviruses in the human genome was aligned to the mouse genome (using the tool LiftOver, *http://genome.ucsc.edu/cgi-bin/hgLiftOver*) after excision of all repetitive elements (performed with RepeatMasker, *http://www.repeatmasker.org/*). Nothing informative was found by measuring the association of mouse H3K4me3 with these syntenic regions and comparing these values with those obtained using control loci.

P300 and H3K27ac bind intra-species conserved regions and co-localize in embryonic-specific enhancers [34,51]. These markers are also associated with HERV-H in human ES cells, lying within 4 kB of 80% of HERV-H proviruses (F score 0.95; p < 10^-300^). It seems unlikely that an exogenous retrovirus would be capable of recruiting these factors by exploiting random conserved regions around its integration site. This suggests that a pre-existing layer of epigenetic markers favored integration of HERV-H into particular host loci and that these features are still preserved millions of years later.

## Conclusions

Among retroelements in the human genome, the endogenous gammaretrovirus HERV-H is extraordinary for its high level expression in embryonic stem cells, in which it makes up 2% of all polyadenylated RNA. The human genome has ~1,000 copies of HERV-H, and the majority of the HERV-H RNA is encoded by a subset of 50 of these. HERV-H expression decreases as ES cells lose pluripotency, to the point where its expression is undetectable in fibroblasts. Consistent with this expression pattern, HERV-H is also expressed to high level in many iPS cells, though expression in some iPS cells is more modest; this heterogeneity may reflect reported differences in the epigenetic profile of many iPS cells, when compared with ES cells [29]. This suggests, then, that HERV-H RNA offers a relatively stringent marker for human pluripotency that would be worth monitoring during the generation of new iPS lines. The HERV-H RNAs in ES cells average about 5 kB in length and encode no protein. It, therefore, seems likely that HERV-H RNA contributes to pluripotency by acting as a chromatin-associated structural element or by acting as a microRNA decoy.

## Methods

### Statistical analysis of association between chromatin markers and retro-elements

Association with a given marker was defined as the presence of the endogenous proviral DNA within a fixed distance (usually 2 kilobases) from the nearest marker on the linear sequence of the chromosome. Unlike exogenous retroviruses, the endogenous virus is already integrated. Therefore, to restore the conditions before integration, the distance from the j-esim marker *M*_*j*_ with peaks in loci {*m*_*j*0_, …, *m*_*jN*_} and the i-esim provirus *V*_*i*_ spanning along the loci {*V*_*i*_^*s*^, *V*_*i*_^*e*^} has been calculated as

$$\begin{array}{l} d\left(V_{i},M_{j}\right)=\min\left(\left|{V_{i}}^{s}-M_{j}\right|,\left|{V_{i}}^{e}-M_{j}\right|\right)\\ \;=\min\left(\left|{V_{i}}^{c}-M_{j}\right|\right)-\frac{V_{i}^{e}-V_{i}^{s}}{2} \end{array}$$

where $V_{i}^{c}=\frac{V_{i}^{s}+V_{i}^{e}}{2}$ is the central locus of the provirus.

As a control dataset, we randomly selected 100000 genomic locations. Association strength was measured with the statistical method based on the F score, as previously described in [18].

Formally the *F*_*β*_-score is defined as the β-weighted harmonic mean of Precision(P) and Sensitivity(R): $F_{\beta }\equiv \left(1+\beta^{2}\right)\frac{\text{PR}}{\beta^{2}P+R}$.

Here β = 0.5 to give more weight to Precision than to Sensitivity. This balances type I and type II errors by adjusting for the high rate of False Positives inherent to the examination of large datasets for genome-wide binding sites according to statistical significance (F score based statistics and comparison with other measures have been extensively discussed in [18]).

Markers with F scores ranging between 0.5 and 1 were considered to be associated with endogenous integration sites.

### RNASeq data analysis

HERV-H is present in more than 1000 “imperfect” copies in the human genome and its transcripts share a number of short conserved regions (each around 100 bp). Therefore, deep sequencing of those transcripts yields reads (25-75 bp long sequences) which perfectly align to several genomic loci. Indeed, multireads mapping is still a challenging process [52]. The strategy adopted here was to perform the alignment of uniquely mapped single- and paired-end reads and to reassign the multiple-mapped reads in function of the expression level of the surrounding (context) region [26]. HERV-H expression was evaluated in term of “Reads Per Kilobase per Million mapped reads (RPKM)” with the standard formula $E_{r}=K\frac{N_{r}}{L_{r}N_{T}}$[53] where *N*_*r*_ is the number of reads mapping onto the *r* transcript, *L*_*r*_ is the length (in kB) of the *resim* transcript and *N*_*T*_ is the total number of reads *K* = 10^6^.

Alignments of RNASeq generated reads have been performed with a two-step procedure. First we used *Bowtie*[54] on raw data, admitting up to two mismatches for each alignment, and then we discriminated between unique mapping reads and multireads, 80% and 20% of the total number of reads respectively. As expected, many multireads matched with repeated elements. Ignoring them would have resulted in a potential underestimation of the expression of endogenous retroviruses and, in general, of all repetitive elements. Therefore, we adopted a probabilistic assignment based on the amount of reads that univocally map onto the surrounding regions (context), as described in more detail in the next paragraph. This evaluation is also useful to establish if a repeated element is expressly transcribed or if it is part of another structure (i.e. a gene).

### Probabilistic alignment of multiple mapping reads

The *uniquely mapping read* was defined as a short sequence generated by high throughput sequencing that can be aligned to a single genomic region *s*. Accordingly, we defined the *multiread* as a sequence *r* that aligns to a set of M regions {S_1_,…,S_M_}. For each region *s*_*i*_ the *context region* is *c*_*i*_ = *C*_*i*_/(*C*_*i*_ ∩ *s*_*i*_) being *C*_*i*_ a genomic region of *n* nucleotides encompassing *s*_*i*_. The assumption that the amount of reads is proportional to the amount of actual mRNA implies that the set of multireads is distributed on the reference genome accordingly to the amount of uniquely mapping reads aligned to the context regions. Consider $R_{D}\left(s\right)$ as the function giving the number of reads of the dataset D that map univocally to the genomic region s described by the tuple (*chr*, *start*, *end*). Therefore, the probability of the read *r* to be actually part of the mRNA generated from the region *s*_*i*_ is estimated as:

$$P_{D}\left(r\in s_{i}\right)=\frac{R_{D}\left(c_{i}\right)}{\sum_{j}^{M}R_{D}\left(c_{j}\right)}.$$

Eventually, the set of multireads mapping to the same M regions is then partitioned to {S_1_,…,S_M_} accordingly to *P*_*D*_ (*s*_*i*_).

### Specificity

The first axiom of high throughput sequencing asserts that the number of reads aligned to a specific genomic region is proportional to how much RNA has been generated by this region within the cell. At this point it is worth observing that repeated element (RE) sequences might be present in the RNA just because they are part of longer mRNAs.

Since we expect that elements having a specific biological function are independently transcribed, we attempted to distinguish between RE specifically expressed with their own promoter from those that are part of longer RNAs. The number of reads mapped to a region *s* can be naively modeled as a linear combination of *specific reads T*(*s*), *unspecific reads U*(*s*) and additional zero-mean *noise* σ^2^ that account for all other experimental and non-systematic fluctuations that can randomly influence the output of the sequencing process. Formally:

$$R\left(s\right)=U\left(S\right)+T\left(s\right)+\sigma^{2}$$

where $R\left(s\right)$, as before, is the function giving the number of reads assigned to the region *s*.

Therefore, the mean number of reads in the region *s* is:

$$E\left[T\left(s\right)\right]=R\left(s\right)-U\left(s\right).$$

In order to estimate *U*(*s*), it is possible to count the number of reads mapped to the context region *c* as previously shown. Therefore we set

$$U\left(s\right)=\frac{R\left(c\right)}{2},$$

and we eventually adopt the following approximation to correct for the non-specificity of transcription:

$$E\left[T\left(s\right)\right]≅R\left(s\right)-\frac{R\left(c\right)}{2}$$

## Abbreviations

HERV: Human endogenous retrovirus; ES: Embryonic stem; iPS: Induced pluripotent stem; LTR: Long terminal repeat; lncRNA: Long non-coding RNA.

## Competing interests

The authors declare that they have no competing interests.

## Authors’ contributions

FS and JL conceived and designed the experiments. FS, JG, and JL analyzed the data. FS and JL wrote the paper. All authors read and approved the final manuscript.

## References

[B1] Stoye JP Studies of endogenous retroviruses reveal a continuing evolutionary saga Nat Rev Microbiol 2012 10 395 406 22565131 10.1038/nrmicro2783

[B2] Weiss RA The discovery of endogenous retroviruses Retrovirology 2006 3 67 10.1186/1742-4690-3-67 17018135 PMC1617120

[B3] Subramanian RP Wildschutte JH Russo C Coffin JM Identification, characterization, and comparative genomic distribution of the HERV-K (HML-2) group of human endogenous retroviruses Retrovirology 2011 8 90 10.1186/1742-4690-8-90 22067224 PMC3228705

[B4] Stengel A Roos C Hunsmann G Seifarth W Leib-Mosch C Greenwood AD Expression profiles of endogenous retroviruses in Old World monkeys J Virol 2006 80 4415 4421 10.1128/JVI.80.9.4415-4421.2006 16611901 PMC1472034

[B5] Mi S Lee X Li X Veldman GM Finnerty H Racie L LaVallie E Tang XY Edouard P Howes S Syncytin is a captive retroviral envelope protein involved in human placental morphogenesis Nature 2000 403 785 789 10.1038/35001608 10693809

[B6] Vargas A Moreau J Landry S LeBellego F Toufaily C Rassart E Lafond J Barbeau B Syncytin-2 plays an important role in the fusion of human trophoblast cells J Mol Biol 2009 392 301 318 10.1016/j.jmb.2009.07.025 19616006

[B7] Mangeney M Renard M Schlecht-Louf G Bouallaga I Heidmann O Letzelter C Richaud A Ducos B Heidmann T Placental syncytins: genetic disjunction between the fusogenic and immunosuppressive activity of retroviral envelope proteins Proc Natl Acad Sci U S A 2007 104 20534 20539 10.1073/pnas.0707873105 18077339 PMC2154466

[B8] Coffin J Hughes S Varmus H Retroviruses 1997 Cold Spring Harbor Laboratory Press, New York 21433340

[B9] Muster T Waltenberger A Grassauer A Hirschl S Caucig P Romirer I Fodinger D Seppele H Schanab O Magin-Lachmann C An endogenous retrovirus derived from human melanoma cells Cancer Res 2003 63 8735 8741 14695188

[B10] Kolson DL Gonzalez-Scarano F Endogenous retroviruses and multiple sclerosis Ann Neurol 2001 50 429 430 10.1002/ana.1235 11601492

[B11] Nexo BA Christensen T Frederiksen J Moller-Larsen A Oturai AB Villesen P Hansen B Nissen KK Laska MJ Petersen TS The etiology of multiple sclerosis: genetic evidence for the involvement of the human endogenous retrovirus HERV-Fc1 PLoS One 2011 6 e16652 10.1371/journal.pone.0016652 21311761 PMC3032779

[B12] Petersen T Moller-Larsen A Thiel S Brudek T Hansen TK Christensen T Effects of interferon-beta therapy on innate and adaptive immune responses to the human endogenous retroviruses HERV-H and HERV-W, cytokine production, and the lectin complement activation pathway in multiple sclerosis J Neuroimmunol 2009 215 108 116 10.1016/j.jneuroim.2009.08.015 19766328

[B13] Karlsson H Schroder J Bachmann S Bottmer C Yolken RH HERV-W-related RNA detected in plasma from individuals with recent-onset schizophrenia or schizoaffective disorder Mol Psychiatry 2004 9 12 13 10.1038/sj.mp.4001439 14571258

[B14] Young GR Eksmond U Salcedo R Alexopoulou L Stoye JP Kassiotis G Resurrection of endogenous retroviruses in antibody-deficient mice Nature 2012 491 774 778 23103862 10.1038/nature11599 PMC3511586

[B15] Garrison KE Jones RB Meiklejohn DA Anwar N Ndhlovu LC Chapman JM Erickson AL Agrawal A Spotts G Hecht FM T cell responses to human endogenous retroviruses in HIV-1 infection PLoS Pathog 2007 3 e165 10.1371/journal.ppat.0030165 17997601 PMC2065876

[B16] SenGupta D Tandon R Vieira RG Ndhlovu LC Lown-Hecht R Ormsby CE Loh L Jones RB Garrison KE Martin JN Strong human endogenous retrovirus-specific T cell responses are associated with control of HIV-1 in chronic infection J Virol 2011 85 6977 6985 10.1128/JVI.00179-11 21525339 PMC3126607

[B17] van der Kuyl AC HIV infection and HERV expression: a review Retrovirology 2012 9 6 10.1186/1742-4690-9-6 22248111 PMC3311604

[B18] Santoni FA Hartley O Luban J Deciphering the code for retroviral integration target site selection PLoS Comput Biol 2010 6 e1001008 10.1371/journal.pcbi.1001008 21124862 PMC2991247

[B19] Santoni FA EMdeCODE: a novel algorithm capable of reading words of epigenetic code to predict enhancers and retroviral integration sites and to identify H3R2me1 as a distinctive mark of coding versus non-coding genes Nucl Acid Res 2012 in press 10.1093/nar/gks1214 PMC3561958 23234700

[B20] Hawkins RD Hon GC Lee LK Ngo Q Lister R Pelizzola M Edsall LE Kuan S Luu Y Klugman S Distinct epigenomic landscapes of pluripotent and lineage-committed human cells Cell Stem Cell 2010 6 479 491 10.1016/j.stem.2010.03.018 20452322 PMC2867844

[B21] Guenther MG Frampton GM Soldner F Hockemeyer D Mitalipova M Jaenisch R Young RA Chromatin structure and gene expression programs of human embryonic and induced pluripotent stem cells Cell Stem Cell 2010 7 249 257 10.1016/j.stem.2010.06.015 20682450 PMC3010384

[B22] Robertson G Hirst M Bainbridge M Bilenky M Zhao Y Zeng T Euskirchen G Bernier B Varhol R Delaney A Genome-wide profiles of STAT1 DNA association using chromatin immunoprecipitation and massively parallel sequencing Nat Methods 2007 4 651 657 10.1038/nmeth1068 17558387

[B23] Bernstein BE Stamatoyannopoulos JA Costello JF Ren B Milosavljevic A Meissner A Kellis M Marra MA Beaudet AL Ecker JR The NIH roadmap epigenomics mapping consortium Nat Biotechnol 2010 28 1045 1048 10.1038/nbt1010-1045 20944595 PMC3607281

[B24] Kunarso G Chia NY Jeyakani J Hwang C Lu X Chan YS Ng HH Bourque G Transposable elements have rewired the core regulatory network of human embryonic stem cells Nat Genet 2010 42 631 634 10.1038/ng.600 20526341

[B25] Lister R Pelizzola M Dowen RH Hawkins RD Hon G Tonti-Filippini J Nery JR Lee L Ye Z Ngo QM Human DNA methylomes at base resolution show widespread epigenomic differences Nature 2009 462 315 322 10.1038/nature08514 19829295 PMC2857523

[B26] Wu JQ Habegger L Noisa P Szekely A Qiu C Hutchison S Raha D Egholm M Lin H Weissman S Dynamic transcriptomes during neural differentiation of human embryonic stem cells revealed by short, long, and paired-end sequencing Proc Natl Acad Sci U S A 2010 107 5254 5259 10.1073/pnas.0914114107 20194744 PMC2841935

[B27] Adli M Zhu J Bernstein BE Genome-wide chromatin maps derived from limited numbers of hematopoietic progenitors Nat Methods 2010 7 615 618 10.1038/nmeth.1478 20622861 PMC2924612

[B28] Mikkelsen TS Ku M Jaffe DB Issac B Lieberman E Giannoukos G Alvarez P Brockman W Kim TK Koche RP Genome-wide maps of chromatin state in pluripotent and lineage-committed cells Nature 2007 448 553 560 10.1038/nature06008 17603471 PMC2921165

[B29] Bock C Kiskinis E Verstappen G Gu H Boulting G Smith ZD Ziller M Croft GF Amoroso MW Oakley DH Reference maps of human ES and iPS cell variation enable high-throughput characterization of pluripotent cell lines Cell 2011 144 439 452 10.1016/j.cell.2010.12.032 21295703 PMC3063454

[B30] Ruthenburg AJ Allis CD Wysocka J Methylation of lysine 4 on histone H3: intricacy of writing and reading a single epigenetic mark Mol Cell 2007 25 15 30 10.1016/j.molcel.2006.12.014 17218268

[B31] Barski A Cuddapah S Cui K Roh TY Schones DE Wang Z Wei G Chepelev I Zhao K High-resolution profiling of histone methylations in the human genome Cell 2007 129 823 837 10.1016/j.cell.2007.05.009 17512414

[B32] Bernstein BE Mikkelsen TS Xie X Kamal M Huebert DJ Cuff J Fry B Meissner A Wernig M Plath K A bivalent chromatin structure marks key developmental genes in embryonic stem cells Cell 2006 125 315 326 10.1016/j.cell.2006.02.041 16630819

[B33] Celniker SE Dillon LA Gerstein MB Gunsalus KC Henikoff S Karpen GH Kellis M Lai EC Lieb JD MacAlpine DM Unlocking the secrets of the genome Nature 2009 459 927 930 10.1038/459927a 19536255 PMC2843545

[B34] Rada-Iglesias A Bajpai R Swigut T Brugmann SA Flynn RA Wysocka J A unique chromatin signature uncovers early developmental enhancers in humans Nature 2011 470 279 283 10.1038/nature09692 21160473 PMC4445674

[B35] Tada T Tada M Toti-/pluripotential stem cells and epigenetic modifications Cell Struct Funct 2001 26 149 160 10.1247/csf.26.149 11565807

[B36] Takahashi K Yamanaka S Induction of pluripotent stem cells from mouse embryonic and adult fibroblast cultures by defined factors Cell 2006 126 663 676 10.1016/j.cell.2006.07.024 16904174

[B37] Yu J Vodyanik MA Smuga-Otto K Antosiewicz-Bourget J Frane JL Tian S Nie J Jonsdottir GA Ruotti V Stewart R Induced pluripotent stem cell lines derived from human somatic cells Science 2007 318 1917 1920 10.1126/science.1151526 18029452

[B38] Chambers I Silva J Colby D Nichols J Nijmeijer B Robertson M Vrana J Jones K Grotewold L Smith A Nanog safeguards pluripotency and mediates germline development Nature 2007 450 1230 1234 10.1038/nature06403 18097409

[B39] Mitsui K Tokuzawa Y Itoh H Segawa K Murakami M Takahashi K Maruyama M Maeda M Yamanaka S The homeoprotein Nanog is required for maintenance of pluripotency in mouse epiblast and ES cells Cell 2003 113 631 642 10.1016/S0092-8674(03)00393-3 12787504

[B40] Boiani M Scholer HR Regulatory networks in embryo-derived pluripotent stem cells Nat Rev Mol Cell Biol 2005 6 872 884 10.1038/nrm1744 16227977

[B41] Boyer LA Lee TI Cole MF Johnstone SE Levine SS Zucker JP Guenther MG Kumar RM Murray HL Jenner RG Core transcriptional regulatory circuitry in human embryonic stem cells Cell 2005 122 947 956 10.1016/j.cell.2005.08.020 16153702 PMC3006442

[B42] Cesana M Cacchiarelli D Legnini I Santini T Sthandier O Chinappi M Tramontano A Bozzoni I A long noncoding RNA controls muscle differentiation by functioning as a competing endogenous RNA Cell 2011 147 358 369 10.1016/j.cell.2011.09.028 22000014 PMC3234495

[B43] Poliseno L Salmena L Zhang J Carver B Haveman WJ Pandolfi PP A coding-independent function of gene and pseudogene mRNAs regulates tumour biology Nature 2010 465 1033 1038 10.1038/nature09144 20577206 PMC3206313

[B44] Tsai MC Manor O Wan Y Mosammaparast N Wang JK Lan F Shi Y Segal E Chang HY Long noncoding RNA as modular scaffold of histone modification complexes Science 2010 329 689 693 10.1126/science.1192002 20616235 PMC2967777

[B45] Tattermusch A Brockdorff N A scaffold for X chromosome inactivation Hum Genet 2011 130 247 253 10.1007/s00439-011-1027-4 21660507

[B46] Chaumeil J Le Baccon P Wutz A Heard E A novel role for Xist RNA in the formation of a repressive nuclear compartment into which genes are recruited when silenced Genes Dev 2006 20 2223 2237 10.1101/gad.380906 16912274 PMC1553206

[B47] Song X Wang B Bromberg M Hu Z Konigsberg W Garen A Retroviral-mediated transmission of a mouse VL30 RNA to human melanoma cells promotes metastasis in an immunodeficient mouse model Proc Natl Acad Sci U S A 2002 99 6269 6273 11959915 10.1073/pnas.092112199 PMC122938

[B48] Meric C Goff SP Characterization of Moloney murine leukemia virus mutants with single-amino-acid substitutions in the Cys-His box of the nucleocapsid protein J Virol 1989 63 1558 1568 2926863 10.1128/jvi.63.4.1558-1568.1989 PMC248388

[B49] Wolf D Goff SP Embryonic stem cells use ZFP809 to silence retroviral DNAs Nature 2009 458 1201 1204 10.1038/nature07844 19270682 PMC2676211

[B50] Wolf D Goff SP TRIM28 mediates primer binding site-targeted silencing of murine leukemia virus in embryonic cells Cell 2007 131 46 57 10.1016/j.cell.2007.07.026 17923087

[B51] Creyghton MP Cheng AW Welstead GG Kooistra T Carey BW Steine EJ Hanna J Lodato MA Frampton GM Sharp PA Histone H3K27ac separates active from poised enhancers and predicts developmental state Proc Natl Acad Sci U S A 2011 107 21931 21936 21106759 10.1073/pnas.1016071107 PMC3003124

[B52] Pepke S Wold B Mortazavi A Computation for ChIP-seq and RNA-seq studies Nat Methods 2009 6 S22 S32 10.1038/nmeth.1371 19844228 PMC4121056

[B53] Mortazavi A Williams BA McCue K Schaeffer L Wold B Mapping and quantifying mammalian transcriptomes by RNA-Seq Nat Methods 2008 5 621 628 10.1038/nmeth.1226 18516045 PMC13303166

[B54] Langmead B Trapnell C Pop M Salzberg SL Ultrafast and memory-efficient alignment of short DNA sequences to the human genome Genome Biol 2009 10 R25 10.1186/gb-2009-10-3-r25 19261174 PMC2690996

